# The Modulatory Effect of Gender and Cytomegalovirus-Seropositivity on Circulating Inflammatory Factors and Cognitive Performance in Elderly Individuals

**DOI:** 10.3390/ijms20040990

**Published:** 2019-02-25

**Authors:** Svetlana Di Benedetto, Marcel Gaetjen, Ludmila Müller

**Affiliations:** 1 Center for Lifespan Psychology, Max Planck Institute for Human Development, Lentzeallee 94, 14195 Berlin, Germany; dibenedetto@mpib-berlin.mpg.de; 2 Center for Medical Research, University of Tübingen, Waldhörnlestr. 22, 72072 Tübingen, Germany; 3 Becton Dickinson Biosciences, Tullastr. 8-12, 69126 Heidelberg, Germany; marcel.gaetjen@bd.com

**Keywords:** aging, immunosenescence, inflammaging, pro-inflammatory cytokines, anti-inflammatory cytokines, cytomegalovirus, gender, cognition

## Abstract

Aging is characterized by a chronic increase in the systemic levels of inflammatory cytokines even in ostensibly healthy individuals. The drivers of age-related increase in systemic inflammation are unclear but one potential contributor may be a persistent infection with Cytomegalovirus (CMV). In this study, we characterized the inflammatory status of 161 older participants recruited to undergo a six-month training intervention. We investigated the influence of gender and CMV-seropositivity on the main inflammatory and anti-inflammatory circulating biomarkers, such as cytokines, receptor antagonist, soluble receptor, immune cells, and relevant metabolic markers. We found that both gender and CMV-seropositivity modulate circulating peripheral biomarkers, and that CMV-infection modifies associations among the latter. Moreover, we observed an interaction between CMV-serostatus and gender associations with cognitive abilities: gender differences in fluid intelligence (Gf) and working memory (WM) were noted only in CMV-negative individuals. Finally, we found that in the CMV-seronegative participants Gf, episodic memory (EM), and WM correlated negatively with pro-inflammatory tumor necrosis factor (TNF); and EM correlated positively with anti-inflammatory interleukin (IL)-10. In CMV-seropositive individuals EM and Gf correlated negatively with pro-inflammatory IL-6, while EM, Gf, and WM correlated negatively with anti-inflammatory IL-1RA. We conclude that both CMV-serostatus and gender may modulate neuroimmune factors, cognitive performance and the relationship between the two domains and should therefore be considered in comparative and interventional studies with elderly people.

## 1. Introduction

Aging has been linked to persistent low-grade systemic inflammation that is characterized by a chronic increase in the levels of circulating pro-inflammatory cytokines, whose presence is highly related to age-related metabolic, cardiovascular, and neuro-degenerative diseases [1]. The disequilibrium between pro- and anti-inflammatory cytokines may have a negative effect on cognitive abilities, inducing learning and memory deficits in Alzheimer’s disease and other neurodegenerative disorders. Although it is unclear even in pathological processes, how systemic inflammation relates to disease processes occurring in the brain, peripheral inflammation and central inflammation may be closely related [2,3]. To underscore the importance of pro- and anti-inflammatory homeostasis in aging, and the role of chronic low-grade inflammation in shaping the aging phenotype, a term “inflammaging” has been coined [4].

Cytokines are signaling molecules possessing unique modulatory functions. They may influence many physiological processes, such as neuroendocrine interactions, neurotransmitter metabolism, and neuroplasticity and affect behavior and cognition [5]. Among numerous pro- and anti-inflammatory cytokines, some stand out as influential contributors to age-related differences in health, immunity, and cognition.

The tumor necrosis factor (TNF) that plays a key role in several neuroimmune functions is associated with the increased risk for neurodegeneration [4,6,7,8]. Through the activation of several pathways, this cytokine contributes to the production of pro-inflammatory interleukin-6 (IL-6) [9,10]. IL-6 that is produced mostly by adipose tissue macrophages, myokines and glial cells is elevated in persons of advanced age and people suffering from obesity. IL-6 may, however, under certain conditions exhibits anti-inflammatory properties through inhibiting TNF release as well as promoting release of anti-inflammatory molecules, such as interleukin 10 (IL-10) [11]. IL-10, an anti-inflammatory cytokine, suppresses, in turn, the release of TNF and other inflammatory cytokines [10,12]. 

Another prominent pro-inflammatory cytokine, interleukin 1 beta (IL-1β) is primarily produced by monocytes, but could also be secreted by myocytes, adipocytes and microglia [13]. The main actions of IL-1β are stimulation of immune cells to produce pro-inflammatory cytokines, activation of microglia, and regulation of neurotrophic activity [14,15]. Alone or in synergy with TNF, IL-1β affects nearly every cell in the organism [13]. Whereas TNF is able to directly activate neurons, IL-1β that shows increased circulating levels with advanced age [12,16], appears to act via microglia [17]. Yet another pro-inflammatory cytokine, interleukin 18 (IL-18) plays an important role in local and systemic inflammation by promoting TNF release and IL-6 production [18]. 

Levels of cytokines can also be affected by the properties of their receptors, and the IL-1 receptor antagonist (IL1-RA) that is secreted by monocytes, neutrophils, and other cells has an important role as an IL-1β receptor blocker, while also acting as a natural inhibitor of TNF. IL-1RA limits or buffers the inflammatory effect of other cytokines [13,19,20,21] and modulates the immune effects of TNF [17]. With so many actors intertwined in multiple synthesis, release and modulation pathways, attributing immune and neural differences to specific cytokines may be a challenging task.

To complicate matters, the interrelated effects of all surveyed cytokines as well as their influence on immune and neuroendocrine functions can be modified by chronic activity of an infectious agent. A lifelong persistent infection influences immunosenescence and can significantly alter the course of cognitive aging when it acts in conjunction with individual differences in cytokine production and release. Currently, consensus seems to be building around the CMV as such a chronic modifier of cytokine action. CMV exerts significant influence on the aging immune system [22,23,24,25] and thus acts as a driving factor of inflammaging [26,27]. Constant immune surveillance and the sustained efforts of the immune system to control reactivations of a dormant virus induce low-level immune response and inflammation, which may have dramatic consequences for health in the elderly [26,28,29,30]. In older adults, CMV has been linked to increased frailty, accelerated cognitive decline, and an increased risk of cardiovascular and Alzheimer diseases [23,26,31,32,33,34,35]. 

To date, very few studies have investigated the associations between CMV and cognition in healthy older individuals and their findings are inconsistent. In a study on 1,061 participants, the CMV-seropositive elderly showed lower cognitive performance than their CMV-seronegative counterparts, and among CMV-infected individuals, higher CMV-antibody levels were associated with lower general cognitive ability [36]. On the other hand, in a cohort of 567 octogenarians, CMV-infection was not associated with functional or cognitive decline [34]. To the best of our knowledge, only one longitudinal study, reported that higher CMV IgG titers were associated with an increased cognitive decline over a 4-year period [35]. 

CMV infection is thought to contribute to many chronic conditions that have been established as predictors of cognitive decline [37,38,39,40], including endothelial dysfunction, dyslipidemia, hypertension, atherosclerosis and coronary heart disease [41,42]. Subclinical inflammation, together with the metabolic and vascular risk factors might be underlying factor for age-related cognitive impairments—with sexual dimorphism additionally influencing these underlying processes. 

Although it is known that the impact of aging on immunity in men and women is different [43,44,45], studies on the influence of gender in humans are still scarce and their results are controversial [43,46,47,48,49]. In our previous study, we have observed multiple gender-related differences in the effects of age and CMV-infection on the differentiation status of T cells [50], suggesting that the CMV-associated “senescence of T cells” in older men may be more pronounced than in older women. Others have later confirmed this effect, which was even found in the middle-aged CMV-positive males and females between 50 and 65 years old [51]. 

The present study posited four major goals. First, we aimed to measure and characterize the baseline inflammatory status of aged individuals recruited for an intervention study of active aging before starting the cognitive and physical training. Specifically, we assessed main inflammatory and anti-inflammatory biomarkers, such as circulating cytokines (IL-1β, TNF, IL-6, IL-10, IL-18), receptor antagonist IL-1RA, and soluble receptor (sTNF-R), immune cells (lymphocytes, leukocytes, monocytes, neutrophils), and relevant metabolic markers: high-density lipoprotein (HDL), low-density lipoprotein (LDL), and triglycerides. Second, we aimed to explore the influence of gender and CMV-seropositivity on the immune and metabolic markers measured at baseline. Third, we examined the associations among inflammatory and metabolic factors, and assessed whether CMV-seropositivity modifies these relationships. Fourth, we explored the influence of the measured inflammatory factors on the cognitive abilities, such as fluid intelligence, episodic memory, speed, and working memory, in the context of CMV-serostatus and gender.

## 2. Results

### 2.1. CMV-Serostatus of Study Participants

**Figure 1:**
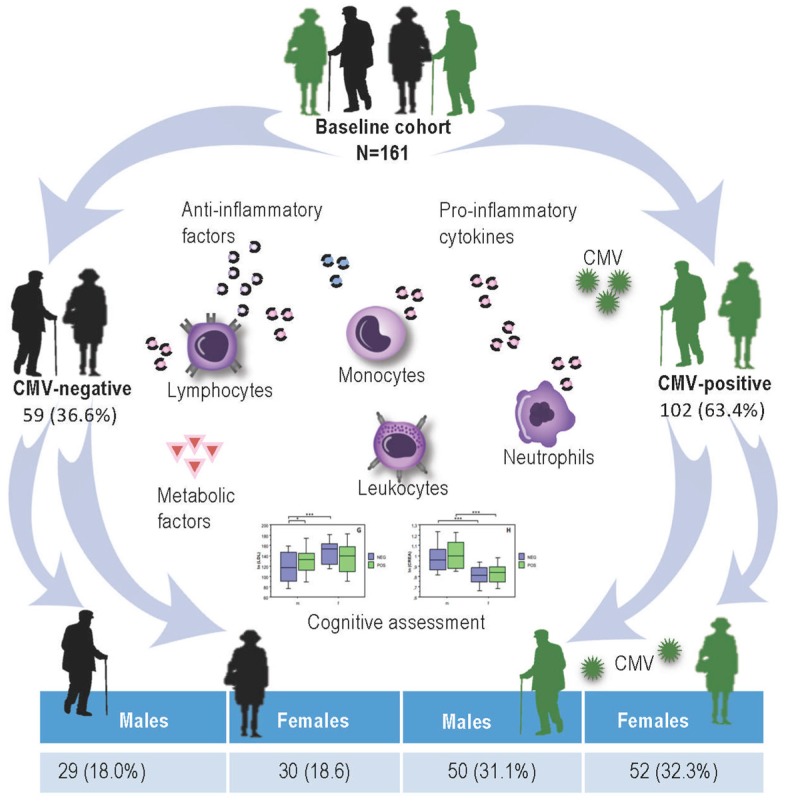
A schematic illustration of the study setup and Cytomegalovirus (CMV)-serostatus of study participants. CMV: Cytomegalovirus.

Results of the CMV-serostatus in both male and female participants are presented in Figure 1. Among 161 participants 102 (63.4%) were CMV-seropositive and 59 (36.6%) CMV-seronegative, whereby CMV-positive group consisted of 50 (31.0%) male and 52 (32.3%) female persons, whereas CMV-negative group contained 29 (18.1%) men and 30 (18.6) women.

### 2.2. Influence of Gender and CMV-Serostatus on Circulating Levels of Pro- and Anti-Inflammatory Mediators, Immune Cells, and Metabolic Blood Values Analysed by MANOVA

For the Multivariate ANOVAs (MANOVA), the logarithmically transformed variables were grouped into pro-inflammatory (IL-1β, IL-6, IL-18, and TNF), anti-inflammatory (IL-10, IL1RA, and sTNF-R), metabolic (HDL, LDL, and triglycerides), and immune cells (lymphocytes, monocytes, and neutrophils) groups of variables. Results of MANOVA, the follow-up univariate ANOVAs, and Scheffé’s post hoc test are described in the following sub-sections accordingly.

#### 2.2.1. Pro- and Anti-Inflammatory Groups of Variables

**Figure 2:**
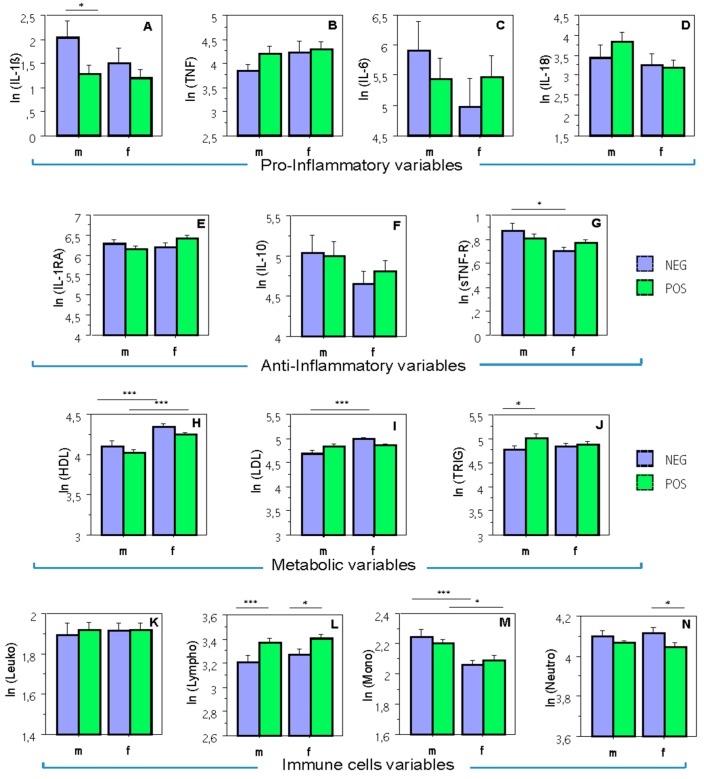
Means and standard errors for all dependent variables by Sex and CMV. Diagrams of concentrations (logarithmically transformed) for: (**A**–**D**) pro-inflammatory variables; (**E**–**G**) anti-inflammatory variables; (**H**–**J**) metabolic variables; (**K**–**N**) immune cells. IL: interleukin; IL-1β: interleukin 1 beta; TNF: tumor necrosis factor; IL-1RA: interleukin 1 receptor antagonist; sTNF-R: soluble tumor necrosis factor receptor; HDL: high-density lipoprotein; LDL: low-density lipoprotein; m: male; f: female; POS: NEG: CMV-seronegative CMV-seropositive; ln: natural logarithm. *, *p* < 0.05; ***, *p* < 0.001.

MANOVA for pro-inflammatory group of variables showed no significant effects for any of the factors or for interaction between them. However, a separate univariate ANOVA on the outcome variable IL-1β revealed a significant effect of CMV-serostatus, F(1,157) = 4.52, *p* < 0.05, whereby according to the Scheffé’s post hoc test, only male subjects showed significant differences: NEG (negative) > POS (positive), mean diff. = 0.77, crit. diff. = 0.72, *p* < 0.05 (Figure 2A).

MANOVA for anti-inflammatory variables showed a significant effect of gender, F(3,155) = 4.16, *p* < 0.01, whereby separate univariate ANOVAs revealed a significant effect of gender for sTNF-R only, F(1,157) = 6.97, *p* < 0.01. As indicated by the Scheffé’s post hoc test, sex differences were significant only in CMV-negative group, mean diff. = 0.17, crit. diff. = 0.13, *p* < 0.05 (Figure 2G).

#### 2.2.2. Group of Metabolic Risk Variables

In the case of the metabolic blood values, MANOVA showed a significant effect of the factor Gender, F(3,155) = 14.85, *p* < 0.0001, and a significant interaction Gender by CMV, F(3,155) = 3.84, *p* < 0.05. Separate univariate ANOVAs revealed a significant effect of the factor Gender for HDL, F(1,157) = 26.39, *p* < 0.0001, and LDL, F(1,157) = 11.63, *p* < 0.001, and a significant interaction Gender by CMV for LDL, F(1,157) = 9.93, *p* < 0.01. As shown by the Scheffè post hoc test, HDL demonstrated significant sex differences in both CMV-negative, mean diff. = 0.23, crit. diff. = 0.13, *p* < 0.01, and CMV-positive participants, mean diff. = 0.22, crit. diff. = 0.11, *p* < 0.0001 (Figure 2H), whereas LDL was higher in female as compared with male subjects only for the CMV-negative group, mean diff. = 0.29, crit. diff. = 0.14, *p* < 0.0001 (Figure 2I). In addition, there was also a significant effect of the factor CMV for HDL, F(1,157) = 4.0, *p* < 0.05, and for Triglycerides, F(1,157) = 6.55, *p* < 0.05. Interestingly, when performing the Scheffé’s post hoc test, the CMV effect for HDL did not reach a significance level either in males or in females, and triglycerides revealed significant differences only in males, mean diff. = 0.24, crit. diff. = 0.22, *p* < 0.05 (Figure 2J).

#### 2.2.3. Group of Immune Cells Variables

As for the immune cells’ group, there were significant effects of the factors Gender, F(4,154) = 4.39, *p* < 0.01, and CMV, F(4,154) = 3.75, *p* < 0.01 found by MANOVA. Separate univariate ANOVAs revealed a significant effect of the factor Gender for monocytes, F(1,157) = 16.07, *p* < 0.0001, and a significant effect of the factor CMV for lymphocytes, F(1,157) = 13.21, *p* < 0.001, and neutrophils, F(1,157) = 6.55, *p* < 0.05. Sex differences for monocytes were significant in both CMV-negative, mean diff. = 0.18, crit. diff. = 0.10, *p* < 0.001 and CMV-positive participants, mean diff. = 0.11, crit. diff. = 0.09, *p* < 0.05 (Figure 2M). CMV differences for lymphocytes were significant in both male participants, mean diff. = 0.16, crit. diff. = 0.11, *p* < 0.01 and female participants, mean diff. = 0.13, crit. diff. = 0.12, *p* < 0.05 (Figure 2L), while these differences for neutrophils were significant only in female participants, mean diff. = 0.07, crit. diff. = 0.06, *p* < 0.05 (Figure 2N).

### 2.3. Influence of Gender and CMV-Serostatus on Circulating Levels of Pro- and Anti-Inflammatory Mediators, Immune Cells, and Metabolic Blood Values Analysed by Bootstrapping Approach

**Figure 3:**
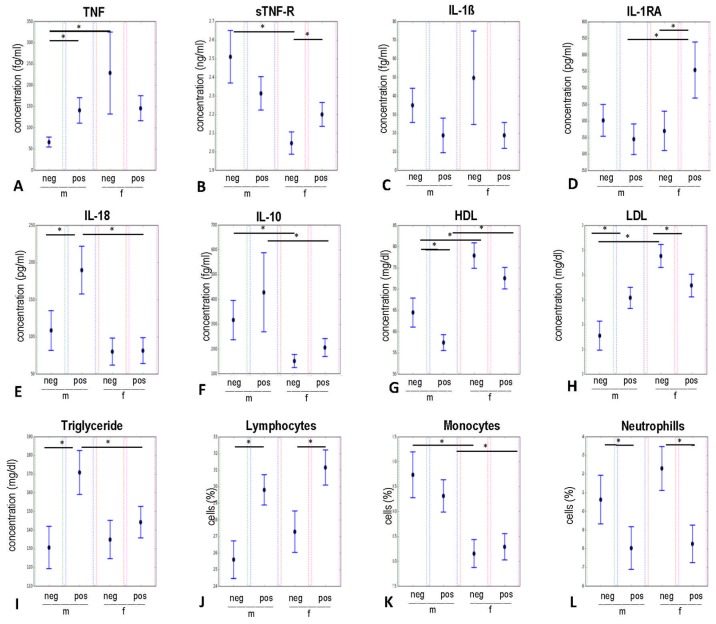
Bootstrapping results on the influence of CMV-serostatus and gender on circulating pro- and anti-inflammatory cytokines, receptor antagonist, immune cells, and metabolic factors. Data are presented as means with 95% confidence intervals for CMV-negative and CMV-positive males as well as for CMV-negative and CMV-positive females. * *p* < 0.05. IL-1β: interleukin 1 beta; IL-1RA: interleukin 1 receptor antagonist; TNF: tumor necrosis factor; sTNF-R: soluble tumor necrosis factor receptor; IL: interleukin; HDL: high-density lipoprotein; LDL: low-density lipoprotein; m: male; f: female; pos: CMV-seropositive; neg: CMV-seronegative.

The results of bootstrapping analyses are presented in Figure 3. As demonstrated by a confidence interval (*CI* = 95%) obtained from bootstrapping, the following significant group differences in mean levels were observed at *p* < 0.05.

The inflammatory TNF (Figure 3A) was significantly increased in the CMV-seropositive group of male participants compared to the CMV-seronegative males. Furthermore, there were clear sex differences in the CMV-seronegative group; namely, female participants showed an increased level of TNF compared to males.

In contrast, the anti-inflammatory sTNF-R (Figure 3B) was increased in males compared to females in the CMV-seronegative group. Furthermore, sTNF-R was significantly increased in the CMV-seropositive females compared to the CMV-negative female participants.

No significant differences between groups were found for IL-1β levels (Figure 3C). The modulating effect of CMV on IL-RA was detected in females (Figure 3D), where CMV-seropositive women produced more of this receptor antagonist than CMV-seronegative women did. Gender also influenced the IL-1RA levels, with females having higher concentrations of this inhibitor compared to males in the CMV-seropositive group.

The inflammatory IL-18 (Figure 3E) was increased in males in relation to females in the CMV-seropositive group. CMV-seropositive male participants also showed increased levels of IL-18 compared to their counterparts from the CMV-seronegative group. Levels of anti-inflammatory IL-10 (Figure 3F) showed gender differences (male > female) in both, CMV-seronegative and in CMV-seropositive groups.

CMV-seropositive males had significantly increased levels of LDL (Figure 3H) compared to CMV-seronegative male participants, whereas the CMV-seropositive females showed decreased levels of LDL compared to uninfected women. The levels of LDL in CMV-seronegative subjects were significantly increased in females compared to males.

We also looked at the differences in the concentration of HDL (Figure 3G) and found increased levels in CMV-seronegative compared to CMV-seropositive male participants. Moreover, women showed a significantly higher concentration of HDL than men, regardless of their CMV-status. Levels of triglyceride (Figure 3I) in the serum of the CMV-seropositive male group were significantly higher compared to the CMV-seronegative men. In the CMV-seropositive group men showed increased levels compared to women.

Concerning the differences in the levels of tested immune cells, we observed that CMV-seropositive men and women showed a decreased percentage of neutrophils (Figure 3L) compared to CMV-seronegative participants. In contrast, the levels of lymphocytes (Figure 3J), were increased in the groups of CMV-seropositive males and females. Additionally, we observed higher proportions of monocytes (Figure 3K) in males than in females, regardless of their CMV-serostatus.

### 2.4. Associations Among Various Blood Biomarkers in the CMV-Negative and CMV-Positive Groups and Modulatory Effect of CMV-Infection on the Correlation Coefficients

**Figure 4:**
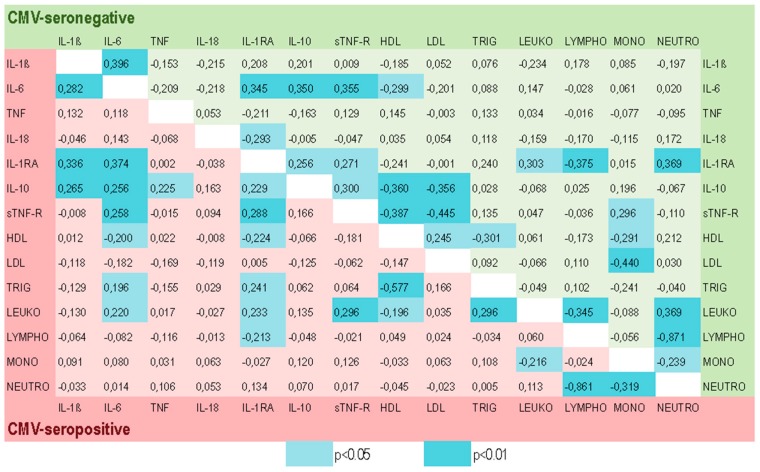
Correlations among different blood biomarkers and the modulatory effect of CMV-seropositivity. Correlation coefficients are presented for CMV-negative in the green field and for CMV-positive in the red field. The significant values are highlighted in light blue (*p* < 0.05) and dark blue (*p* < 0.01). IL-1β: interleukin 1 beta; IL-1RA: interleukin 1 receptor antagonist; IL: interleukin; TNF: tumor necrosis factor; sTNF-R: soluble tumor necrosis factor receptor; HDL: high-density lipoprotein; LDL: low-density lipoprotein; LYMPHO: lymphocytes; LEUKO: leukocytes; MONO: monocytes; NEUTRO: neutrophils.

Figure 4 presents the Pearson’s correlation coefficients for both the CMV-seropositive (red) and the CMV-seronegative (green) groups. Correlations’ significance values are not adjusted for multiple comparisons [52].

We found that the correlations seen in the CMV-seronegative and CMV-seropositive groups of participants were different concerning their magnitude. Below we describe results of the additionally applied Steiger’s procedure showing the significant differences in the correlation coefficients between these two groups. We found that the correlation between the pro-inflammatory cytokine TNF and the anti-inflammatory cytokine IL-10 was significantly increased in the CMV-positive compared to the CMV-negative group (Zd = 0.394, *p* < 0.05). A similar significant increase in the CMV-positive compared to the CMV-negative group was found between the correlation coefficients of triglyceride and leukocytes (Zd = 0.353, *p* < 0.05), and between triglyceride and HDL (Zd = −0.347, *p* < 0.05).

In contrast, the relationship between anti-inflammatory sTNF-R and cholesterol LDL was significantly decreased (Zd = 0.416, *p* < 0.05) in the CMV-positive compared to the CMV-negative elderly participants. Furthermore, the magnitude of the correlation coefficients between monocytes and LDL (Zd = 0.535, *p* < 0.01), and between cholesterols LDL and HDL (Zd = −0.397, *p* < 0.05) were significantly lower under influence of the CMV-infection.

### 2.5. The Modulatory Effect of the CMV-serostatus and Gender on the Cognitive Abilities of Study Participants

The confirmatory factor analyses (CFA) of the four latent cognitive factor model (episodic memory, working memory, fluid intelligence, and perceptual speed) resulted in a good fit, χ^2^48 = 55.4; CFI = 0.99; RMSEA = 0.036; SRMR = 0.047 (Figure S1 in the supplemental material).

**Figure 5:**
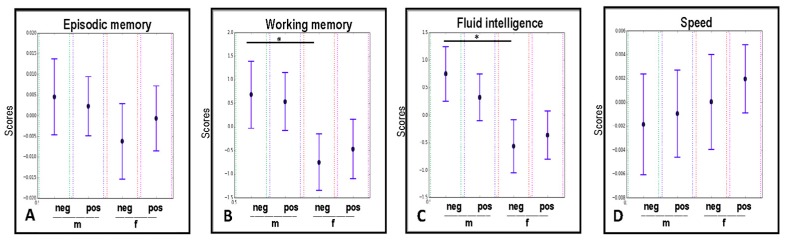
The influence of CMV-serostatus and gender on episodic memory, working memory, fluid intelligence, and processing speed, analyzed with the Bootstrapping approach. * *p* < 0.05. m: male; f: female; pos: CMV-seropositive; neg: CMV-seronegative.

Figure 5A–D shows bootstrapping results on the effects of gender and CMV infection on cognitive abilities of study participants. We found that both these factors had modulatory effects on fluid intelligence measurements, where male individuals showed significantly higher scores of fluid intelligence compared to females, but only in the group of CMV-seronegative participants (Figure 5C).

The same phenomenon was observed also for the working memory domain (Figure 5B). Results obtained for episodic memory, and perceptual speed showed no significant differences in any of the tested groups (Figure 5A,D).

### 2.6. Associations Between Cognition and Circulating Inflammatory Mediators

To investigate potential associations between circulating pro- and anti-inflammatory biomarkers and cognition, we assessed correlations between scores of cognitive performance and inflammatory cytokines (TNF and IL-6), soluble receptor (sTNF-R), and receptor antagonist, IL-1RA. Table 1 summarizes the relationships between these variables. We found that in the CMV-seronegative participants, episodic and working memory as well as fluid intelligence correlated negatively with pro-inflammatory TNF levels. Episodic memory demonstrated a positive association with anti-inflammatory IL-10.

In the CMV-seropositive elderly, fluid intelligence and episodic memory correlated negatively with pro-inflammatory IL-6, but also with anti-inflammatory IL-1RA. Similarly, the working memory showed negative associations with IL-1RA in the CMV-positive individuals.

The correlations seen in the CMV-seronegative and CMV-seropositive groups of participants were found to be different concerning their magnitude. To test for the significant differences in the correlation coefficients between these two groups, we applied Steiger’s procedure. We found that the correlation between pro-inflammatory cytokine TNF and fluid intelligence was significantly increased (Zd = 0.387, *p* < 0.05) in the CMV-positive group compared to the CMV-negative group. In contrast, the relationships between IL-1RA and episodic memory (Zd = −0.445, *p* < 0.01), and between IL-1RA and working memory (Zd = −0.379, *p* < 0.05) were significantly decreased in the CMV-positive compared to the CMV-negative elderly participants.

## 3. Discussion

**Figure 6:**
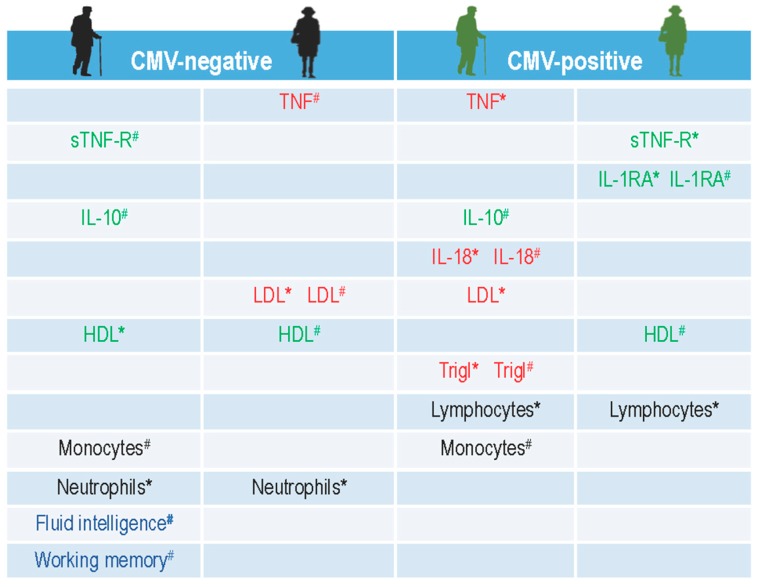
A schematic illustration of summarized results on the influence of gender and CMV-serostatus on circulating pro- and anti-inflammatory cytokines, receptor antagonist, metabolic factors, immune cells, and cognitive abilities in the baseline cohort of elderly participants. The names of the analyzed parameters with significantly higher values are placed in the corresponding column of the CMV-negative or CMV-positive men and women accordingly, whereby the notation (^#^) implies: a gender-related-higher value, and the notation (*) implies: a CMV-related-higher value. The pro-inflammatory mediators are written in red; the anti-inflammatory are green; the cognitive latent factors are blue; and immune cells are left in black. TNF: tumor necrosis factor; sTNF-R: soluble tumor necrosis factor receptor; IL: interleukin; IL-1β: interleukin 1 beta; IL-1RA: interleukin 1 receptor antagonist; HDL: high-density lipoprotein; LDL: low-density lipoprotein.

In the present study, we characterized the inflammatory status of aged individuals at the baseline in a pre-intervention cohort. In a first set of analyses, we investigated the influence of gender and CMV-seropositivity on the main inflammatory and anti-inflammatory mediators and molecules assessed in this study, such as circulating cytokines, receptor antagonist, soluble receptor, immune cells, and relevant metabolic markers. We found that both gender and CMV-seropositivity jointly and separately participate in the modulation of circulating pro- and anti-inflammatory biomarkers in elderly study participants. Figure 6 illustrates the summarized results of these effects.

The influence of sexual dimorphism on the inflammatory status was demonstrated in both CMV-negative and CMV-positive participants. While in the CMV-seronegative group, males demonstrated significantly higher levels of anti-inflammatory sTNF-R, the females had elevations of its pro-inflammatory counterpart, TNF, probably, due to the missing anti-inflammatory effects of sTNF-R in their circulation. The level of the anti-inflammatory receptor antagonist, IL-1RA, was, in contrast, significantly higher in CMV-positive women than in CMV-positive men. This deficit possibly contributed to an increase in the pro-inflammatory IL-18 in men due to the property of IL-1RA to propagate and to buffer the effect of inflammatory cytokines [13,19,20,21].

The influence of gender on the cytokine profile in elderly humans has rarely been studied and findings are contradictory. Some studies have shown gender-related differences [53], whereas others found no differences for the majority of cytokines [12]. The in vitro evidence suggests that testosterone may suppress the production of the pro-inflammatory cytokines TNF, IL-1β, and IL-6 [54], and potentiate the release of the anti-inflammatory cytokine IL-10 [55]. Our results are mainly congruent with these findings, showing a significant gender-related increase in levels of anti-inflammatory IL-10 in men of both CMV-positive and CMV-negative groups. Pro-inflammatory TNF is also lower in men compared to women, but this concerns only the CMV-negative groups. It seems that the CMV-seropositivity might possibly diminish the positive effect of testosterone on the inflammatory status in aged males, because not only elevations of pro-inflammatory TNF and IL-18, but also decreased levels of anti-inflammatory sTNF-R and IL-1RA have been found in CMV-infected male participants (Figure 6).

In our previous publication, we assumed that “gender disparities in the differentiation status of immune cells might first emerge under the immunomodulating effect of the stress of long-term immunosurveillance to control CMV-infection” [50]. The same might also be true for circulating cytokines as well, not least because circulating inflammatory molecules are, in fact, mostly produced by the same senescent CMV-exhausted immune cells. Such senescent cells secrete various extracellular factors, including inflammatory cytokines, which can enhance and “propagate senescence with autocrine and paracrine modality, contributing to the pro-inflammatory status of ageing” [56].

The percentage of immune cells in our baseline cohort appeared to be also modulated by CMV-serostatus, with increased levels of lymphocytes and decreased proportions of neutrophils in CMV-positive old participants. At the same time, blood monocytes vary in our sample by gender, with men having higher proportion of monocytes compared to women, similar to what was reported in earlier studies [57]. These aging immune cells and particularly, the inflammatory fraction of monocytes, are thought to be responsible for inflammation-induced “unhealthy” aging [19].

Intriguing results on higher levels of anti-inflammatory mediators such as IL-1RA and sTNF-R in CMV-positive as compared to CMV-negative women on the one hand, and higher pro-inflammatory state in CMV-negative women as compared to CMV-negative men on the other hand (Figure 6) may be due to the participants’ chronological history of co-existence with the CMV-antigen [58,59] and to the sex-specific differences in the immune responses [60,61]. Generally, immune responses in females are characterized by more pronounced pro-inflammatory activation that is partly regulated by estrogen receptors (ER). ERs form complexes at gene regulatory elements and promote epigenetic changes and transcription, thereby regulating inflammatory response in a dose- and context-dependent manner. Low physiological levels of estradiol generally promote inflammatory pathways leading to production of pro-inflammatory cytokines. In some conditions, however, ER signaling inhibits these pathways even in a low estrogen environment [62]. Such a special condition may represent the latent CMV infection, which may persist on a lifelong basis.

One of the possible explanations might be that the initial immune response of young women to a primary CMV infection could be different to young men’s (due to the less active immunity in the latter) and that this initial difference (together with other factors) translates to an induction of a pro-inflammatory environment in the aged CMV-positive men, while in the post-menopausal CMV-positive women, this may perhaps lead to generation of anti-inflammatory mediators instead. However, clearly more in-depth studies are required, including investigations on the impact of modulatory effects of sex hormones on immune interactions, to further define and to clearly delineate these effects.

Inflammation can also adversely affect lipoproteins, which may then, in turn, modulate the production of pro-inflammatory cytokines [63]. Results from the animal studies demonstrated that inflammatory cytokines such as TNF, IL-1β, and IL-6 increase serum triglyceride fatty acid levels [63,64]. We have also found a positive association between levels of pro-inflammatory IL-6 and triglyceride, but exclusively in the CMV-positive participants (Figure 4). Our results show also multiple significant associations of HDL- and LDL-cholesterols with pro- and anti-inflammatory cytokines and their receptors (Figure 4). Moreover, the strength of these associations seems to be modified by CMV infection. Additionally, the inflammatory environment appeared to be less pronounced in the groups with higher concentrations of serum HDL (Figure 6).

In general, HDL has potent anti-inflammatory properties and the remarkable ability to modulate the inflammatory response in various cell types. However, in a chronic inflammatory state, HDL can itself be modified to become dysfunctional [65] and therefore not able to relieve cells from excessive and oxidized LDL cholesterol. The most common effects of a chronic pro-inflammatory state are decreases in serum HDL and increases in triglycerides, total cholesterol, and LDL. Thus, in addition to affecting serum lipid levels, inflammation also adversely affects the lipoprotein function [66,67]. Our results showed similar effects of increased LDL and triglycerides concentrations, but decreased HDL levels in the inflammatory environment of CMV-negative females and CMV-positive males (Figure 6). Moreover, it appears that both CMV-seropositivity and gender jointly (increase of LDL in CMV-negative women; increase of triglycerides in CMV-positive men) and separately (decrease of HDL in CMV-positive men) contributed to these effects. However, it is clear that more studies involving different pro- and anti-inflammatory biomarkers and their modulators are required to understand these multifactorial and dynamic interrelationships and their effects on low-grade inflammation and immunosenescence.

In the present study, we assessed the cognitive performance of elderly people at baseline of a six-month intervention study. Moreover, we investigated the influence of the CMV-serostatus and gender on cognitive abilities. Here, we found that gender exerted a modulating effect on the fluid intelligence and working memory (with men showing higher scores of performance) in the CMV-negative individuals only, whereas no such influence has been detected for processing speed and episodic memory in any of the tested groups (Figure 5).

Gender differences in cognitive tests have repeatedly been reported in older adults although the magnitude of these differences seems to be modest [68] and advantage for one or another gender appears to be related to different cognitive domains [69]. Gender differences in cognitive test performance have been attributed to various factors, such as sex hormones or sexual dimorphisms in brain structure—all of which change with normal aging [70], but the modulating influence of CMV-seropositivity has not been investigated in these studies. Al-Delaimy demonstrated higher cognitive test performance among men, but not among women and these differences were related to insulin-like growth factor (IGF)-1 levels [71]. Similar results on the IGF-1 that positively influenced the cognitive performance only in men, were also shown by another group [72]; and, again, the influence of CMV was not considered in this study.

In the current study, males from the CMV-negative group demonstrated better scores in fluid intelligence, working memory and concomitantly, a high level of peripheral anti-inflammatory factors, such as IL-10 cytokine and soluble TNF receptor (Figure 6). Furthermore, they also showed higher levels of anti-inflammatory HDL compared to males of the CMV-seropositive group. Interestingly, CMV-positive men did not show such cognitive advantage, although their levels of IL-10 were higher than in CMV-positive women. This could partly be explained by the fact that they had an elevated inflammatory status (CMV-related higher levels of TNF and IL-18), a relatively adverse metabolic environment (elevated LDL cholesterol and triglycerides), and increased levels of monocytes and lymphocytes in their peripheral circulation (Figure 6). All these factors are known to contribute to a low-grade inflammation and some of them, acting as upstream effectors might also mediate effects of peripheral inflammation on the central nervous system and have powerful effects on cognition and behavior [5,73,74]. Therefore, we can speculate that the integral effect of the above-described conditions in their peripheral circulation might influence and modulate cognitive abilities of elderly people.

In our study, we have demonstrated a negative association of fluid intelligence as well as episodic and working memory with the pro-inflammatory TNF in CMV-negative individuals. TNF is known to exert physiological neuroprotective but also pathological neurodegenerative effects [75] within the nervous system. Cognitive impairments have also been demonstrated in transgenic mice over-expressing TNF [76]. The pro-inflammatory TNF and IL-1β have been shown to physiologically modulate synaptic plasticity and synaptic scaling in different brain areas such as hippocampus, striatum and cortex [77,78].

In contrast, we found a positive association of episodic memory with anti-inflammatory IL-10 in the CMV-negative group. IL-10 is known for its inhibitory role on the production of inflammatory cytokines by microglia as well as for its neuroprotective function on neurons and astrocytes [79].

Interestingly, in the CMV-seropositive group, fluid intelligence, episodic and working memory scores were negatively associated with the anti-inflammatory IL-1RA, the levels of which were apparently simultaneously increased as a reaction to the rise of the pro-inflammatory cytokines in their periphery. This phenomenon has also been observed by other groups [80,81], who reported that individuals with elevated levels of the pro-inflammatory markers also tend to show increased levels of the anti-inflammatory markers. In the CMV-positive group we have also found a negative associations of episodic memory and fluid intelligence with pro-inflammatory IL-6. Several mediators of inflammatory activity, including such cytokines as IL-6, IL-1β, and TNF, were shown to be associated with impairments in the cognitive function [82,83,84,85]. Our results on the negative association of the cognitive performance with inflammatory cytokines are in accordance with these findings.

In our investigation of the relationships between various inflammatory biomarkers, we have also found that the CMV-latency influenced interrelations between the different mediators of inflammation, possibly contributing to the induction of such a CMV-related inflammatory environment. In other words, the CMV-infection appears not only to contribute to the shift in the levels of the particular cytokine but also to the change in the interrelationships between these immune mediators and molecules. Due to the exploratory character of the study on these associations, more in-depth investigations are required to confirm and elucidate these associations and altered interrelationships between different biomarkers under the modulatory influence of CMV-infection.

Findings from a comprehensive study that aimed to create a source of immune measurements in aging individuals including among others, clinical and functional parameters, peripheral blood mononuclear cells (PBMC) phenotypes, cytokines and gene expression in stimulated and unstimulated PBMC, as well as measures of some serum cytokines, showed that age, followed by sex and CMV status had the greatest effect on the immune system [86].

Thus, we can conclude that the CMV-latency may induce various modulatory effects on the inflammatory and immune factors in the peripheral circulation of aged individuals. This modulatory activity may have different consequences for the aged men and women and, therefore, may also differently influence their functional and cognitive abilities. On account of this, both the CMV-serostatus and gender should always be included in the consideration together with other factors in the comparative and interventional studies with elderly people.

Our study has many strengths, including that it is one of the first studies to extensively characterize prior to physical, cognitive, and combine interventions, the inflammatory and functional status in elderly participants by accessing the multiple pro- and anti-inflammatory cytokines, receptor antagonist, soluble receptor, metabolic factors, immune cells, and multiple measures of objective cognitive function. This is also one of the first studies to assess the modulatory effect of CMV-seropositivity and gender on the inflammatory status of participants and their functional cognitive abilities at baseline.

There are several limitations in our study that should be acknowledged. The first one is related to the fact that our pre-training cohort consisted of relatively healthy, non-obese, and well-educated Berlin residents with a comparatively low seroprevalence for CMV for this age. For this reason, the generalizability of some of our findings may be limited to the Berlin healthy aging population or to a similar European population in urban areas.

Another limitation may be related to the fact that we did not evaluate the serostatus of the study participants for other chronic or latent infections, such as EBV (Epstein-Barr virus), HIV (Human Immunodeficiency virus), HBV (Hepatitis B virus), or HCV (Hepatitis C virus), to confirm that the observed results are specifically related to CMV infection.

The next limitation that has been repeatedly reported also by several other studies [12,81,87,88,89], may be due to the fact that such cytokines as IL-1β, TNF, and IL-6 are not highly abundant in the periphery of relatively healthy non-obese people, and the levels of such cytokines were also found in some of our participants towards the lower end or below the levels of detection for these assays. Accordingly, a further limitation may be related to the sensitivity of the techniques used to detect cytokines. The most frequently applied quantification of cytokine levels using Enzyme-linked Immunosorbent Assay (ELISA) technique may sometimes not be sensitive enough, due to the “presence of naturally occurring biological inhibitors in circulation, which might interfere with the detection of the respective cytokine” [90]. Also, multiplex techniques and even the Cytometric Bead Array (CBA) Enhanced Sensitivity Flex Set used in our study “are primarily designed to accommodate the simultaneous measurement of several analytes, and therefore compromises are inevitably made for the individual analytes” [91]. Despite these limitations, results obtained in the present study for the most of the pro- and anti-inflammatory cytokines and other factors related to low-grade inflammation are rather consistent.

While the interaction of pro- and anti-inflammatory cytokines, receptor antagonist, soluble receptor, and metabolic factors are complex and still need to be understood in the context of age-related low-grade inflammation, our results suggest that the evaluation of both gender differences and the impact of the CMV-serostatus appears to be decisive in studies dealing with age-related changes in neuroimmune factors as well as their association with cognitive and behavioral abilities in elderly people.

## 4. Materials and Methods

### 4.1. Participants

The sample consisted of 161 older adults (Figure 1) who had enrolled to participate in a training study that included physical, cognitive, and combined training interventions. Male and female subjects were recruited from volunteer participants pools at the Max Planck Institute for Human Development and by advertisements in the metropolitan area of Berlin, Germany. All the volunteers lived independently at home, leading an active life. Participants were healthy, right-handed adults, aged 64–79 years. All volunteers completed a medical assessment prior to data collection. The medical examination was conducted at the Charité Sports Medicine, Charité Universitätsmedizin Berlin. Of the originally recruited 201 volunteers only 179 individuals met inclusion criteria for study participation after medical assessment. None of the participants had a history of head injuries, medical (e.g., heart attack), neurological (e.g., epilepsy), or psychiatric (e.g., depression) disorders. None of the volunteers had suffered from chronic inflammatory, autoimmune or cancer disease, nor had clinically evident infections. Moderately elevated and controlled blood pressure was not considered as exclusion criteria. All subjects completed the informed consent form to the study protocol which was approved by the Ethics Committee of German Society of Psychology on 27.09.2016, UL 072014.

### 4.2. Circulating Biomarkers Assessment

#### 4.2.1. Cytokines TNF, IL-10, IL-6, and IL-1β

The serum levels of pro- and anti-inflammatory cytokines (TNF, IL-10, IL-6, and IL-1β) were determined using the high-sensitivity cytometric bead array (CBA) flex system (BD Biosciences, San Jose, CA, USA) that allows quantification of the serum concentration of these inflammatory markers in a single sample. All analyses were performed according to the manufacturer’s instructions; to increase accuracy an additional standard dilution was added. The fluorescence produced by CBA beads was measured on a BD FACS CANTO II Flow Cytometer and analyzed using the software FCAP Array v3 (BD Biosciences).

#### 4.2.2. sTNF-R, IL-1RA, IL-18 Levels, and CMV-Serostatus

To gauge sTNF-R (80 kDA), IL-1RA, and IL-18 levels, we used the Sandwich Enzyme-linked Immunosorbent Assay (ELISA), a sensitive method allowing for the measurement of an antigen concentration in an unknown sample. All analyses were conducted according to the manufacturer’s instructions. The levels of human circulating sTNF-R (80 kDA), IL-1RA, and IL-18 were determined using the Platinum ELISA kit for the quantitative detection of the three cytokines (ThermoFisher SCIENTIFIC Invitrogen, Vienna, Austria, catalog numbers: BMS211, BMS2080 and BMS267/2).

Serum levels of the Cytomegalovirus IgG were determined using the commercial ELISA kit (IBL International GMBH, Hamburg, Germany, catalogue number: RE57061) and according to the manufacturer’s instructions. Samples were considered to give a positive signal if the absorbance value exceeded 10% over the cut-off, whereas a negative signal was declared if the absorbance value was lower than 10% below the cut-off.

All samples were assessed in duplicates at 450 or 450/620 nm using Multiscan-FC Microtiter Plate Photometer. Protein concentrations were determined in relation to a four-parameter standard curve (Prism 8 GraphPad, San Diego, CA, USA) or calculated using Microsoft Excel 2011.

Levels of LDL- and HDL-cholesterols, triglyceride, lymphocytes, leukocytes, monocytes, and neutrophils were measured within the clinical diagnostics facility of Berlin, Labor28. Serum concentrations of cholesterols and triglyceride were measured using enzymatic colorimetric tests (Roche, Basel, Switzerland). The counts of the immune cells were assessed by flow cytometry (Sysmex, Norderstedt, Germany).

### 4.3. Cognitive Assessment

Participants were invited to one session that lasted about 3.5 h. Participants were tested in groups of four to six. The cognitive battery included a broad range of measures of learning and memory performance, processing speed, working memory, and executive functioning. The group received a standardized session protocol and started, after instructions, with a practice trial to ensure that all participants understood the task. Responses were collected via button boxes, the computer mouse, or the keyboard.

For the purpose of the present study, we focused on four latent factors representing main cognitive abilities, namely episodic memory (EM; measured by Verbal Learning and Memory Test, Face–Profession Task, and Scene Encoding), working memory (WM; measured by Letter Updating, Number-N-Back, and Spatial Updating), fluid intelligence (Gf; measured by Figural Analogies, Letter Series, and Practical Problems), and perceptual speed (Speed; measured by Verbal Speed, Figural Speed, and Number Speed) [92,93,94,95,96]. The detailed description of the factors and tasks is included in the supplementary material.

### 4.4. Statistical Analyses

The participants were split into two groups, depending on their CMV-serostatus: CMV-seropositive and CMV-seronegative and further divided by gender. All variables distributions were examined for normality using the Kolmogorov-Smirnov and Shapiro–Wilk tests. Because variables were significantly departed from normality and exhibited variance heterogeneity, the natural logarithm transformation was applied. For cytokine levels below the detection range of the assay sensitivity, the LOD/square root of 2 (where LOD is the lowest level of detection) was used [97,98,99].

To investigate the influence of CMV-serostatus and gender on circulating pro- and anti-inflammatory biomarkers, immune cells, and metabolic factors, the MANOVA and bootstrapping analyses were performed. For MANOVA, the logarithmically transformed variables were grouped into pro-inflammatory (IL-1β, IL-6, IL-18, and TNF), anti-inflammatory (IL-10, IL-1RA, and sTNF-R), metabolic (HDL, LDL, and triglycerides), and immune cells (lymphocytes, monocytes, and neutrophils) groups of variables. Further, follow-up univariate ANOVAs were performed to investigate the influence of CMV-serostatus and gender on the single outcome variables. Scheffé’s post hoc test was used to determine which of the paired means differed significantly.

For the bootstrap approach [100] we used untransformed data. Bootstrapping generated different samples with similar distributions and provided estimates of confidence intervals around sampling means. The procedure involved drawing 10,000 samples with replacement from a single original sample in four groups (CMV^−^ males; CMV^−^ females; CMV^+^ males; CMV^+^ females), calculating statistics for each sample, and inspecting the bootstrap distribution of the re-sampling means. Since the bootstrap distribution showed a normal shape and a small bias, we could obtain a 95% confidence interval (*CI*) for the mean by using the bootstrap standard error (*SE_boot_*) and the *t* distribution: *CI* = *mean* ± *t* × *SE_boot_*, using LabView software with the MatLab bootstrap function. The level of statistical significance was set at *p* < 0.05.

To investigate the relationship between the levels of inflammatory and anti-inflammatory cytokines, immune cells counts, and metabolic blood characteristics, we calculated Pearson’s correlations for CMV-seropositive and CMV-seronegative groups separately. Correlation analyses were performed with logarithmically transformed data. Since it was an exploratory study, analyses were performed without adjustment for multiple comparison [52].

A test for significant differences in the correlation coefficients was performed using Steiger’s method [101]. To test the null hypothesis that the correlation between two variables in one sample is the same as the correlation between these variables in another sample, we first, carried out Fisher’s Z transform for each of the two correlation coefficients:

(1) $$Z=0.5\times ln\left(\frac{1+r}{1-r}\right)$$

and then calculated the test statistic (*Z_d_*) as follows:

(2) $$Z_{d}=\frac{Z_{1}-Z_{2}}{\sqrt{\frac{1}{n_{1}-3}+\frac{1}{n_{2}-3}}}$$

where *n*_1_ and *n*_2_ are corresponding sample numbers.

A Confirmatory Factor Analyses of four latent cognitive factors was applied to define a four-factor model of cognitive abilities in latent space by using measures of specific cognitive tasks as manifest variables. The four intercorrelated latent factors of cognitive constructs were as follow: working memory (WM), episodic memory (EM), fluid intelligence (Gf), and processing speed (Speed). All latent factors were allowed to be correlated (Figure S1 in Supplementary Materials).

## 5. Conclusions

In the present study we found that both gender and CMV-seropositivity modulate circulating peripheral biomarkers, and that CMV infection modifies associations among the latter. Moreover, we observed an interaction between CMV-serostatus and gender associations with cognitive abilities: Gender differences in fluid intelligence and working memory were noted only in CMV-negative individuals. Finally, we found that in the CMV-seronegative participants fluid intelligence, episodic memory, and working memory correlated negatively with pro-inflammatory TNF. We also found that episodic memory correlated positively with anti-inflammatory IL-10. In CMV-seropositive individuals, episodic memory and fluid intelligence correlated negatively with pro-inflammatory IL-6; and episodic memory, fluid intelligence, and working memory correlated negatively with anti-inflammatory IL-1RA. We conclude that both CMV-serostatus and gender may modulate neuroimmune factors, cognitive performance and the relationship between the two domains and should therefore be considered in comparative and interventional studies with elderly people.

## Figures and Tables

**Table 1:** Pearson’s correlation coefficients (*r*) and corresponding *p*-values for associations between cognitive performance scores and circulating peripheral inflammatory markers.

Variables	*r*	*p*-Value
CMV-negative group (*n* = 59):		
EM ^1^ vs. TNF	−0.330	0.010
EM vs. IL-10	0.282	0.029
WM vs. TNF	−0.334	0.009
Gf vs. TNF	−0.415	0.001
CMV-positive group (*n* = 102):		
EM vs. IL-6	−0.201	0.042
EM vs. IL-1RA	−0.214	0.030
WM vs. IL-1RA	−0.218	0.028
Gf vs. IL-6	−0.205	0.039
Gf vs. IL-RA	−0.272	0.005

^1^ EM. episodic memory; WM: working memory; Gf: fluid intelligence; TNF: tumor necrosis factor; IL: interleukin; IL-1RA: interleukin 1 receptor antagonist; CMV: Cytomegalovirus.

## Supplementary Materials

Supplementary materials can be found at https://www.mdpi.com/1422-0067/20/4/990/s1.

## Abbreviations

CMV	Cytomegalovirus
IL	Interleukin
IL-1RA	Interleukin 1 receptor antagonist
TNF	Tumor Necrosis Factor
sTNF-R	Soluble Tumor Necrosis Factor receptor
HDL	High-density lipoprotein
LDL	Low-density lipoprotein
IgG	Immunoglobulin G
ANOVA	Analysis of variance
MANOVA	Multivariate ANOVAs
CI	Confidence interval
EM	Episodic memory
WM	Working memory
Gf	Fluid intelligence
CFA	Confirmatory Factor Analysis
IGF-1	Insulin-like growth factor 1
ELISA	Enzyme-linked Immunosorbent Assay
CBA	Cytometric bead array
