# Supplementary material for: The Modulatory Effect of Gender and Cytomegalovirus-Seropositivity on Circulating Inflammatory Factors and Cognitive Performance in Elderly Individuals
Source: Int J Mol Sci. 2019 Feb 25;20(4):990. doi: 10.3390/ijms20040990 (PMC6412896; doi:10.3390/ijms20040990)
Supplement: Supplementary file 1 [file ijms-20-00990-s001.pdf]

Supplementary materials

Episodic memory

Verbal Learning and Memory Test (List). This task assesses auditory verbal learning of 15 words including five learning trials, early recall, an interference list after five learning trials, free recall tests directly after an interference list as well as 30 min later (late recall), and a final recognition test (Helmstaedter and Durwen 1990). The sum of items recalled across trials 1 to 5 provides a measure of overall learning performance that was used here as the manifest variable.

Face–Profession task (Face). This task assesses associative binding on the basis of recognition of incidentally encoded face–profession pairs. During the study phase 45 face–profession pairs were each presented for 3.5 s on the computer screen and the participants had to indicate via button presses whether the faces matched the profession or not. After a 3-min delay between study and test phase 54 face–profession pairs consisting of 27 old pairs, 9 new pairs, and 18 newly arranged pairs were presented (in newly arranged pairs the shown face is the same, but is associated with a new profession). The participants were asked to decide whether they had seen a given face–profession combination before or not and to rate the confidence of their decision on a three-point scale ranging from 1 = not sure to 3 = very sure. Recognition memory for the rearranged face–profession pairs (hit minus false alarms) served as the manifest variable in the model.

Object Location task (Object). In this task, sequences of 12 colored photographs of real-world objects were displayed at different locations in a 6-by-6 grid. After presentation, objects appeared at the side of the screen and had to be moved to the correct locations by clicking on the objects and the locations with the computer mouse. One practice trial and two test trials were included. The sum of correct placements across the two test trials is used as the manifest variable.

Working memory (WM)

Spatial Updating (Spatial). In each block of this task, a display of two or three 3-by-3 grids was shown for 4 s. In each of these, one blue dot was presented in one of the nine locations. Those two or three locations had to be memorized and updated according to shifting operations that were indicated by arrows appearing below the corresponding field. The presentation time of the arrows was 2.5 s with an inter-stimulus interval of 0.5 s. After six updating operations, the two or three grids reappeared and the resulting end positions had to be clicked on. After ten practice blocks with memory loads of two and three grids, ten test blocks with load two and three were conducted and used for scoring. The average percentage of correct placements was used as one of the manifest variables for the working memory (WM) factor (Schmiedek et al. 2010).

Letter Updating (LetterU). In this task subjects were presented with 7, 9, 11, or 13 letters in a sequence. Once a sequence stopped, subjects had to report the last three letters in correct order by pressing buttons on the button box corresponding to A, B, C, and D.

Number-N-Back (Number). Three one-digit numbers (ranging from 0 to 9) were presented sequentially in three cells situated horizontally followed by the next sequence of three digits. This cycle was repeated 30 times. In each cycle, two-choice decisions on whether the current stimulus matched the stimulus shown three steps earlier had to be made. Four practice trials including 30 runs were followed by 6 test trials with 30 runs. Subjects made their decision via button-box presses with their left and right index fingers (Schmiedek et al. 2010).

Fluid intelligence (Gf)

Practical Problems (Problem). This task consisted of 12 items depicting everyday problems such as the times in a bus schedule, instructions for medication, a warranty for a technical appliance, a rail map, as well as other forms and tables. For each item, the problems were presented in the upper part of the screen, and five alternative responses were shown in the lower part. Subjects responded by clicking on one of the five alternatives with the computer mouse. A single practice item was provided. The test phase was terminated when subjects made three consecutive errors, or when they reached the maximum time limit of 10 minutes, or after they had answered the last test item. Items were ordered by difficulty (Lindenberger et al. 1993).

Figural Analogies (Analog). Items in this test followed the format "A is to B as C is to ?". One figure pair was presented in the upper left part of the screen and a single figure was shown beside it. Participants had to use the same rule as the one applying to the complete figure pair to choose one of the five alternative responses presented below. Subjects entered their response by clicking on one of the five alternatives with the mouse. Before the test phase, instructions and three practice items were given. The test phase was terminated when subjects made three consecutive errors, when they reached the maximum time limit (10 min), or after they had worked on the last test item. Items were ordered by difficulty (Lindenberger et al. 1993).

Letter Series (Letter). The task consisted of 22 items. Each item contained five letters followed by a question mark (e.g., c e g i k ?). Items were displayed in the upper half of the screen, and five response alternatives were presented in the lower half. Items followed simple rules such as +1, –1,+2, or +2 +1. Subjects entered their response by touching one of the five answer alternatives. The score was based on the total number of correct responses. Instructions and three practice items were given before the test phase. The test phase was terminated when subjects made three consecutive false responses, when they reached the maximum time limit (6 min), or after they had answered the last item of the test. Items were ordered by difficulty. Sample items were used with respect to tests related to speed, reasoning, and knowledge (Lindenberger et al. 1993).

Processing Speed / comparison task (Speed)

For *the numerical version (number)* of the comparison task, two strings of five numbers each appear on the left and right of the screen, with participants having to decide as quickly as possible whether both strings are exactly the same or different. If different, the strings differ by just one number. Number strings are randomly assembled using digits 1–9.

The verbal version (verbal) of this task is equivalent to the numerical one, using strings of five consonants.

Figural version (figure). Two "fribbles" - the three-dimensional colored objects consisting of several connected parts, are shown to the left and right of the screen, with participants having to decide as quickly as possible whether the two objects are exactly the same or different. If different, the objects differ with respect to one part. The Fribble images in this task are courtesy of Michael J. Tarr, Brown University, http://www.tarrlab.org/). In the session, two trials of 40 items were included for each of the verbal, numerical, and figural tasks.

Confirmatory factor analyses of four latent cognitive factors

CFA was applied to define a four-factor model of cognitive abilities in latent space using measures of specific cognitive tasks selected from the cognitive battery as manifest variables. The model shown in figure S1 fits the data well ($\chi48 = 55.4$; CFI = 0.99; RMSEA = 0.036; SRMR = 0.047).

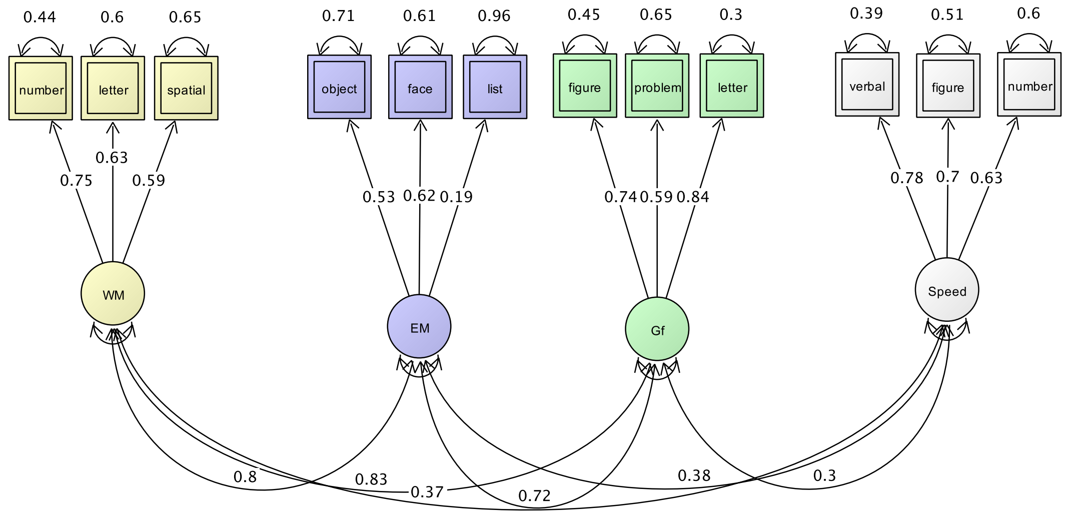

Figure S1. Confirmatory factor analysis (CFA) of four cognitive abilities, working memory (WM), episodic memory (EM), fluid intelligence (Gf), and processing speed (Speed).

Note. Manifest variables loading on each of the latent factors are represented by squares and are z-transformed to ease model convergence. Numbers next to single-headed arrows represent significant standardized path coefficients, and numbers next to double-headed arrows refer to standardized covariances between the latent factors.

Table S1. Descriptive statistics

N=162	**EM**	**WM**	**GF**	**SPEED**
MEAN	.00000	-.00267	-.00536	.00000
MEDIAN	.00578	.54705	.66160	.00523
SD	.052434	4.181651	2.990641	.022990
RANGE	.274	19.386	13.763	.135
MINIMUM	-.153	-11.455	-8.340	-.100
MAXIMUM	.121	7.932	5.424	.035

References:

Chavance M, Escolano S, Romon M, Basdevant A, de Lauzon-Guillain B, Charles MA (2010) Latent variables and structural equation models for longitudinal relationships: an illustration in nutritional epidemiology. BMC Med Res Methodol 10:37. doi:10.1186/1471-2288-10-37

Helmstaedter C, Durwen HF (1990) [The Verbal Learning and Retention Test. A useful and differentiated tool in evaluating verbal memory performance]. Schweiz Arch Neurol Psychiatr (1985) 141 (1):21-30

Lindenberger U, Mayr U, Kliegl R (1993) Speed and intelligence in old age. Psychol Aging 8 (2):207-220

Schmiedek F, Lovden M, Lindenberger U (2010) Hundred Days of Cognitive Training Enhance Broad Cognitive Abilities in Adulthood: Findings from the COGITO Study. Front Aging Neurosci 2. doi:10.3389/fnagi.2010.00027
